# Cryopreserved embryo replacement is associated with higher birthweight compared with fresh embryo: multicentric sibling embryo cohort study

**DOI:** 10.1038/s41598-019-49708-7

**Published:** 2019-09-16

**Authors:** Margaux Anav, Simon Phillips, Alice Ferrieres-Hoa, Anna Gala, Alice Fournier, Claire Vincens, Emmanuelle Vintejoux, Elsa Maris, Camille Grysole, François Bissonnette, Sophie Brouillet, Isaac Jacques Kadoch, Samir Hamamah

**Affiliations:** 1 Univ Montpellier, ART/PGD Department, Montpellier University Hospital, Arnaud de Villeneuve hospital, Montpellier, France; 2 Univ Montpellier, IRMB, INSERM U1203, Montpellier, France; 3 Department of Obstetrics and Gynecology, OVO Fertility, Montreal, Canada; 4 Department of Obstetrics and Gynecology, Montpellier University Hospital, Arnaud de Villeneuve hospital, Montpellier, France; 5 Université Grenoble-Alpes, Centre Clinique et Biologique d’Assistance Médicale à la Procréation-CECOS, Grenoble, France

**Keywords:** Biological techniques, Developmental biology

## Abstract

Birth weight (BW) is higher after frozen embryo transfer (FET) than after fresh embryo replacement. No study has compared the BW of siblings conceived using the same oocyte/embryo cohort. The aim of this study was to determine whether the freezing-thawing procedure is involved in such difference. Multicenter study at Montpellier University Hospital, Clinique Ovo, Canada and Grenoble-Alpes University Hospital. The first cohort (Fresh/FET) included *in vitro* fertilization (IVF) cycles where the older was born after fresh embryo transfer (n = 158) and the younger after transfer of frozen supernumerary embryos (n = 158). The second cohort (FET/FET) included IVF cycles where older and younger were born after FET of embryos from the same cohort. The mean adjusted BW of the FET group was higher than that of the fresh group (3508.9 ± 452.4 g *vs* 3237.7 ± 463.3 g; p < 0.01). In the FET/FET cohort, the mean adjusted BW was higher for the younger by 93.1 g but this difference is not significant (3430.2 ± 347.6 g *vs* 3337.1 ± 391.9 g; p = 0.3789). Our results strongly suggest that cryopreservation is directly involved in the BW variation. Comparing BW difference between Fresh/FET cohort and FET/FET one, it suggests that parity is not the only responsible, increasing the role of cryopreservation step in BW variation.

## Introduction

The first live births after *in vitro* fertilization (IVF) and frozen embryo transfer (FET) were reported in 1978 and 1984 respectively^1,2^. Since then, the use of assisted reproduction technologies (ART) has risen substantially^3^. Relevant perinatal outcome indicators, such as the APGAR score^4^, pH and lactate measurement^5^, and birth weight (BW) are in relation to the baby’s future health. Low BW (LBW) has been associated with the development of chronic diseases such as cardiovascular diseases, hypertension and type 2 diabetes^6^.

In 2018, Maheshwari *et al*. concluded that compared to fresh embryo transfer, FET decreases the risk of LBW, of being small for gestational age and of preterm delivery^7^. In a retrospective cohort study on sibling pairs, Pinborg *et al*. showed that the sibling born after FET has an increased risk of being larger for gestational age than the sibling born after fresh embryo transfer. This demonstrated that intrinsic maternal factors are not the only reasons of the BW difference^8^. Determining the consequences of cryopreservation is crucial because embryo freezing has become a widespread ART procedure^3^.

The aim of this study was to determine in a sibling embryo cohort whether the freezing-thawing procedure is involved in the BW difference between singletons born after fresh embryo transfer and after FET.

Differently from previous studies, here only siblings conceived from the same cohort of embryos (same IVF cycle) were included.

## Material and Methods

### Study design

This multicenter retrospective cohort study at the ART/PGD Department of Montpellier University Hospital, France, Clinique Ovo, Montreal, Canada, and Grenoble-Alpes University Hospital, France. It included all sibling pairs (n = 158) born from the same embryo cohort. In all pairs, the first sibling was born after fresh embryo replacement and the second after FET (both singleton births). It also included all sibling pairs (n = 25) from the same embryo cohort born after FET (singleton births).

To our knowledge, this is the first large observational study investigating the effect of cryopreservation procedure on BW in a sibling embryo cohort.

### Study population and data collection

This study included a first cohort (fresh/FET) with sibling pairs where fresh embryo replacement resulting in a singleton live birth (fresh group, n = 158) was followed by transfer of one or two frozen embryos from the same cohort that resulted in a singleton live birth (FET group, n = 158), between May 2007 and June 2015. The second cohort (FET/FET) included all sibling pairs where FET resulting in a singleton live birth (FET1 group, n = 25) was followed by transfer of one or two frozen embryos from the same cohort that resulted in a singleton live birth (FET2 group, n = 25). Twin pregnancies and stillbirths were excluded. If the BW of at least one sibling was unknown, the pair was not included in the study. This study was approved by local institutional review boards (2019_IRB-MTP_07-01 and OVO R&D scientific comitee) and all participants signed written informed consent. All methods were performed in accordance with the relevant guidelines and regulation.

### IVF procedures

#### Fresh embryo replacement

Patients were treated either with a long stimulation protocol in which after a gonadotropin-releasing hormone (GnRH) agonist, recombinant follicle-stimulating hormone (FSH) or human menopausal gonadotropin (hMG) was administered to stimulate multiple follicle development, or with a short protocol in which recombinant FSH or hMG was administered at day 2 of the cycle followed by a GnRH antagonist. 36 hours after human chorionic gonadotropin (HCG) injection, ultrasound-guided oocyte retrieval was carried out.

One or two fresh embryo(s) was (were) transferred from day 2 to 5 (Table 1). The number of embryos and the choice of the replacement day were based on the woman’s age, rank attempt and embryo quality. Luteal support consisted of daily vaginally administered progesterone.

**Table 1:** Number of embryos transferred and stage of embryo transfer in Fresh/FET cohort and in FET/FET cohort.

	Number of embryos transferred		Stage of embryo transfer		
	1	2	Day 2/3	Day 4	Day 5/6
Fresh (n = 158)	121	37	87	2	69
FET (n = 158)	134	24	62	0	96
FET1 (n = 25)	17	8	13	0	12
FET2 (n = 25)	21	4	12	0	13

In the Fresh/FET cohort, 83 patients received a long stimulation protocol and 75 received a short protocol. In the fresh group, 121 single embryo transfer and 37 double embryo transfer were performed at day 2 (n = 13), 3 (n = 74), 4 (n = 2) or 5 (n = 69) (Table 1).

#### Frozen embryo transfer (FET)

Extra embryos with good morphological aspects were cryopreserved at day 2 or 3 or at day 5 or 6 if the expansion grade was at least 3, the trophectoderm grade A and B, and inner cell mass grade A, B and C according to Gardner’s classification^9^. The cryopreservation procedure was slow freezing until 2011 and then vitrification. In the FET group, embryos were cryopreserved at day 2 (n = 3), day 3 (n = 59), and day 5 or 6 (n = 96) (Table 1).

For 33 patients, embryos frozen were performed by slow freezing and for 125 patients by vitrification.

In the FET/FET cohort, 6 embryos were cryopreserved by slow freezing and 44 by vitrification.

In the FET group, one (n = 134) or two (n = 24) embryos were transferred during a spontaneous cycle if the patient presented regular ovulatory cycles (n = 38), or during an artificial cycle with estrogens and progesterone (n = 93), or s stimulated cycle (n = 27).

For the FET1 group there were 17 single-embryo transfers and 8 double-embryo transfers at day 3 (n = 13) or 5 (n = 12) (Table 1) during a spontaneous cycle (n = 7), artificial cycle (n = 14), or stimulated cycle (n = 4). For the FET2 group, one (n = 21) or two (n = 4) embryos were transferred at day 3 (n = 12), 5 (n = 11) or 6 (n = 2) (Table 1) during a spontaneous cycle (n = 6), artificial cycle (n = 15), or stimulated cycle (n = 4).

#### Slow embryo freezing and thawing

Embryos were incubated in two successive cryoprotectant solutions (1,2-propanediol and sucrose) to obtain a progressive and complete dehydration. The base medium for all freezing solutions was Cryo-PBS (Freeze-Kit 1^TM^, Vitrolife). Embryos were first incubated in Cryo-PBS containing 1.5 M 1,2-propanediol for 10 min, followed by Cryo-PBS with 1.5 M 1,2-propanediol and 0.1 M sucrose. Embryos were then placed in plastic straws and transferred into an automated freezing machine (Cryopreservation Minicool 40PC, Air liquide) at 23 °C. The temperature was progressively reduced to −8 °C at a rate of −2 °C/min and seeding was induced manually in proximity of liquid nitrogen. Straws were cooled to −30 °C at a rate of −0.3 °C/min and then to −150 °C at a rate of −50 °C/min. Straws were then transferred into a liquid nitrogen tank for long-term storage.

For thawing, straws were first warmed at room temperature for few seconds and then immersed in the following cryoprotectants solutions to rehydrate embryos (Thaw-Kit 1^TM^, Vitrolife): 1.0 M 1,2-propanediol + 0.2 M sucrose (5 min), 0.5 M 1,2-propanediol + 0.2 M sucrose (5 min) and 0.2 M sucrose (10 min).

#### Embryo vitrification

Embryos were vitrified using the following: medium 199 (M199)-based solutions (Vitrification Freeze Kit, Irvine Scientific): 7.5% dimethyl sulfoxide (DMSO) + 7.5% ethylene glycol (EG) + 20% dextran serum substitute (DSS; equilibration solution) and 15% DMSO + 15% EG + 20% DSS + 0.5 M sucrose (vitrification solution). Embryos were first placed in the equilibration solution at room temperature for 8–10 min and then in the vitrification solution for 30 s. Embryos were then placed in cryotips and transferred to liquid nitrogen tanks for long-term storage. Cryotips were placed in the M199-based thawing solution (1 M sucrose + 20% DSS). Cryoprotectants were progressively removed using the following M199-based dilution and washing solutions: 0.5 M sucrose + 20% DSS (dilution solution) and 20% DSS (washing solution).

### Statistical analysis

Continuous variables are presented as means and standard deviations. Discrete variables are reported as counts and percentages. In each group (fresh, FET, FET1 and FET2), a multivariate linear regression model was used to adjust the BW to the maternal characteristics (age, body mass index, number of embryos transferred, day of embryo transfer) and perinatal outcomes (gestational age at birth, sex, birth order). The covariates included in the multivariate linear regression model were selected using univariate analyses with level of significance at p ≤ 0.15.

The KruskalWallis test was used to compare the adjusted BW values. Risk differences were reported as adjusted odd ratios (AORs) with 95% confidence intervals (95% CIs). Statistical analyses were performed using the SAS software 9.3 (SAS Institute, Cary, N.C.), and P ≤ 0.05 was considered significant.

## Results

After multivariate analysis, maternal age, body mass index, number and stage of the transferred embryos, tobacco exposure during pregnancy, occurrence of gestational diabetes, sex and parity were not confounding factors in this study. The only factor with a significant effect on BW was gestational age (Table 2). Given the study design, maternal age was higher for singletons born after FET than after fresh embryo transfer and the birth order was always higher for children in the FET group (Table 3).

**Table 2:** *p* of Pearson test correlation between maternal and neonatal caractéristics with BW in ART and natural conception.

	ART	Natural conception
	*p*-values	Impact (+) or no (−)
Maternal age	0.8714	+
Maternal body mass index	0.0516	+
Tobacco exposure	0.9222	+
Occurrence of gestationnel diabete	0.9067	+
gestational age	0.0046	+
Parity	0.8324	+
Sex	0.3018	+
Number of embryos transferred	0.5475	
Stage of embryo transfer	0.5275	
Slow freezing or vitrification	0.4404

**Table 3:** Maternal characteristics and perinatal outcomes in singleton sibling pair in fresh/FET and FET/FET cohort.

Fresh/FET cohort				FET/FET cohort			
	Fresh embryo transfer (n = 158)	FET (n = 158)	*p*-values		FET1 (n = 25)	FET2 (n = 25)	*p*-values
Maternal age (years), mean (SD)	31.4 (4.07)	33.9 (4.03)	<0.0001	Maternal age (years), mean (SD)	32.8 (5.29)	34.9 (5.39)	0.1748
Tobacco exposure (%)	2	3.9	0.5625	Spontaneous cycle (%)	28	24	0.8543
Occurrence of gesational diabete (%)	13.7	16.2	0.7515	Artificial cycle (%)	56	60	0.8543
Male sex (%)	46.2	53.16	0.2171	Stimulated cycle (%)	16	16	0.8543
Gestational age (weeks), mean (SD)	38.6 (2.01)	38.39 (1.5)	0.2961	Male sex (%)	44	32	0.3924
Preterm birth (%)	5.06	8.23	0.4412	Gestational age (weeks), mean (SD)	38.56 (1.39)	38 (1.15)	0.1275
Very preterm birth (%)	2.53	0.63	0.4412	Preterm birth (%)	12	12	1
Birthweight (g), mean (SD)	3247.09 (506.13)	3499.46 (468.26)	<0.0001	Birthweight (g), mean (SD)	3371.44 (436.37)	3395.88 (373.62)	0.8325
Number of embryos transferred	1.25 (0.46)	1.16 (0.4)	0.0929	Number of embryos transferred	1.36 (0.57)	1.2 (0.5)	0.2961

Before adjustment, the mean BW was higher after FET compared with fresh embryo transfer (3499.5 ± 468.3 g *vs* 3247.1 ± 506.1 g; p < 0.0001), conversely, no difference was detected for the FET/FET cohort (Table 3). The mean number of transferred embryos, the occurrence of gestational diabetes, tobacco exposure during pregnancy, sex ratio, gestational age, preterm, and very preterm births were not different between groups in the two cohorts (Table 3).

For the FET/FET cohort, the endometrial preparation was not different in the two groups (Table 3). The age difference between the older and the younger child was 13 to 75 months in the Fresh/FET cohort, and 14 to 62 months in the FET/FET cohort.

**Figure 1:**
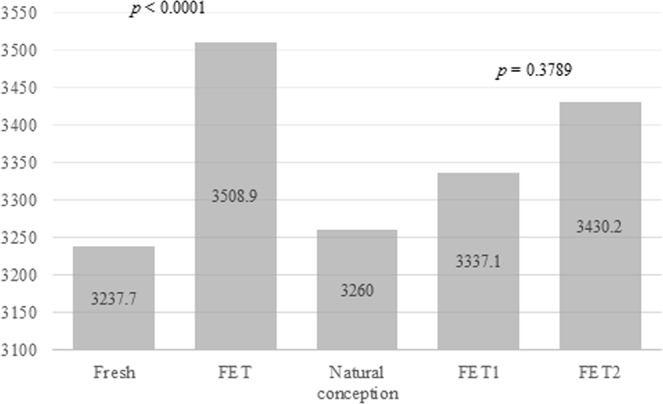
Adjusted birth weight (g) after fresh embryo transfer and frozen embryo transfer in Fresh/FET cohort and in FET/FET cohort, mean BW after natural conception.

The mean adjusted BW in the FET group was significantly higher by 271.2 g than that in the fresh embryo transfer group (3508.9 ± 452.4 g *vs* 3237.7 ± 463.3 g; p < 0.0001) (Fig. 1). This difference remained, regardless of the number of transferred embryos, stage of transfer, and freezing method (slow freezing or vitrification) (Table 4). After adjusting for confounding factors, the risk to be large for gestational age (>90th percentile) was higher in the FET than fresh group (AOR 4.22; 95%CI 2.04–8.73) (Table 5).

**Table 4:** Birthweight after Fresh embryo transfer and frozen embryo transfer in grams (SD) in Fresh/FET cohort according to number of embryos transferred, stage of embryo transfer and freezing method.

	Fresh group (n = 158)	FET group (n = 158)	*p*-values
**Number of embryos transferred**			
1	3254.3 (470.6)	3514.2 (460.7	<0.0001
2	3183.4 (440.5)	3478.8 (410.3)	0.0102
**Stage of embryo transfer**			
Day 2/3	3214.2 (489.7)	3481.8 (464.2)	0.0009
Day 5/6	3269.5 (434.3)	3526.4 (446.1)	0.0003
**Freezing method**			
Slow freezing	3214.8 (389.3)	3549.3 (449.3)	0.0019
Vitrification	3243.7 (482.2)	3498.2 (454.4)	<0.0001

**Table 5:** Risk of being born small for gestational age (SGA) and large for gestational age (LGA) in singleton sibling pairs for second child in Fresh/FET cohort and in FET/FET cohort. AORs (95%CI).

	AOR in Fresh/FET cohort	95% CI	AOR in FET/FET cohort	95% CI
LGA (>90th percentile)	4.22	2.04–8.73	6	0.72–49.84
SGA (<10th percentile)	0.083	0.01–0.64	—	—

In the FET/FET cohort, the mean adjusted BW was higher (by 93.1 g) for the younger child (FET2) (3430.2 ± 347.6 g *vs* 3337.1 ± 391.9 g; p = 0.3789) (Fig. 1) and the risk to be large for gestational age was increased for the younger (AOR 6; 95%CI 0.72–49.84) (Table 5) but in both cases the difference was not significant.

## Discussion

This is the first study that compared the BW after fresh and frozen transfer of embryos from the same embryo cohort. Our findings show that BW is significantly higher in the FET than in the fresh embryo transfer group.

The causes of such difference remain unclear. However, it cannot be explained by intrinsic factors because BW was compared between singleton siblings coming from the same oocyte/embryo cohort and the same parents.

Singletons conceived by double-embryo transfer are more likely to have low BW compared to those conceived by single-embryo transfer^10^. However, in our study, it did not appear to be a confounding factor, although the mean number of transferred embryos was higher in the fresh than in the FET group.

Babies in the FET group were born after a previous successful pregnancy with fresh embryo transfer. Consequently, a parity effect could be involved, as observed in natural conceptions where BW increases with parity^11^. However, Pinborg *et al*. found that in a sibling cohort, the risk of large gestational weight for age is higher after FET compared with fresh embryo transfer even when it was the first^8^. Moreover, the 271.2 g difference in BW between fresh and FET groups far exceeds the 81 g difference due to parity in ART^12^. In agreement, the adjusted BW difference (93.1 g) between FET1 and FET2 groups was not significant. These data indicate that although parity plays a role in BW difference probably it is not the only or the main parameter.

We could also hypothesize that in the FET group, the intrauterine environment was more favorable to embryo growth because it was not affected by controlled ovarian stimulation treatments in comparison with fresh embryo transfer. Such stimulation could negatively affect the peri-implantation uterine environment by reducing the endometrial and subendometrial blood flow^13^, advancing endometrial maturation^14^, or altering the expression profile of genes involved in endometrial receptivity^15–17^. In agreement, Pereira *et al*. recently showed that supraphysiological concentrations of estradiol are an independent predictor of LBW in full-term singletons born after fresh embryo transfer^18^. However, Pinborg *et al*. found that the risk of large gestational weight for age is higher after FET compared with fresh embryo transfer and also natural conception^8^. In our study, BW after FET was even higher than the mean BW after natural conception in France between 2005 and 2013 (i.e., 3260 g ± 451.4 g)^19^ (Fig. 1). We supposed that the intrauterine environment couldn’t be better during FET than in natural conception. Therefore, there is probably another factor to consider.

**Figure 2:**
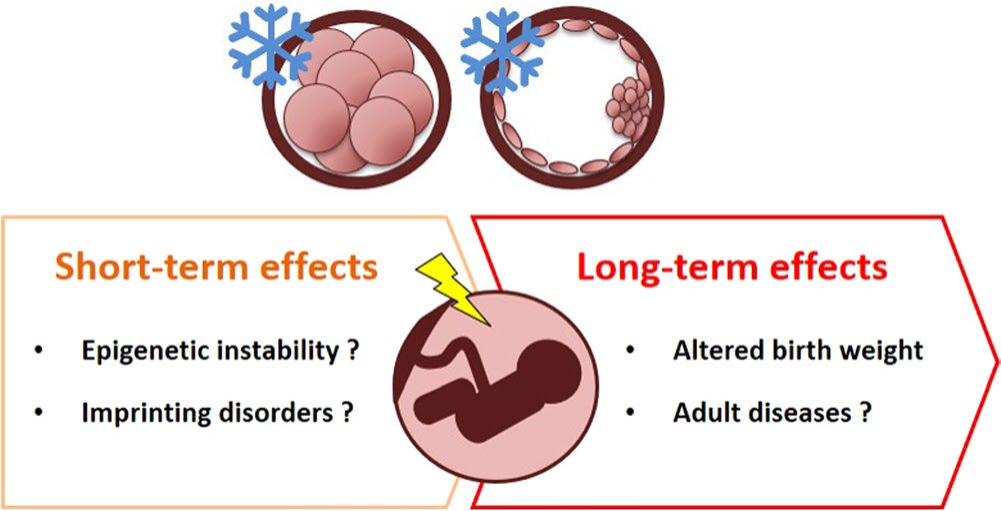
Proposed model of cryopreservation-mediated effects in human embryo and fetal development.

Another likely explanation for the cryopreservation-induced effect on BW could be that embryo freezing leads to epigenetic disturbances that might affect the developmental programming of fetal and placental tissues (Fig. 2). Early embryo development is vulnerable to epigenetic dysregulation^20^ and this stage coincides with the ART treatment period. Animal studies have shown that preimplantation embryo culture affects the methylation profile and expression of imprinted genes^21–24^. These results describe the effects of embryo culture on gene expression, epigenetic regulation and BW; however, it is reasonable to suppose that ART techniques, including embryo freezing/thawing, also could lead to human epigenetic alterations in the same way as in animals. For instance, Miles *et al*. found that children born after IVF are taller and with higher insulin growth factor 1 and 2 (IGF1 and IGF2) levels^25^. As the insulin-IGF system has a crucial role in fetal growth regulation, higher BW could be the result of epigenetic changes after IVF with altered methylation of genes involved in growth and metabolism^25^. Beckwith-Wiedemann Syndrome (BWS) is a disorder in which epigenetic (70% of cases) or genetic alterations lead to excessive growth during the second half of pregnancy and in the first few years of life^26^. In a review from 2013, Vermeiden and Bernardus evaluated that BWS is significantly associated with ART with a pooled relative risk of 5.2 (95% CI 1.6, 7.4)^27^. In the continuity, a recent meta-analysis identified a strong positive association between a history of conception following ART and four imprinting disorders among which BWS with a relative risk of 5.8 (95% CI 3.1–11.1)^28^. In a troubling way, a recent systematic review demonstrated that the combined odds ratio of any imprinting disorder in children conceived by ART is 3.67 (95% CI 1.39–9.74), when compared with naturally conceived children^29^.

In conclusion, our study shows that the BW difference between siblings born after FET and fresh embryo transfer is not due to intrinsic differences and strongly suggests that cryopreservation affects BW through not yet determined mechanisms. On the same line, a recent multicenter randomized controlled trial in women with polycystic ovary syndrome observed a trend toward higher neonatal death after FET compared with fresh embryo replacement^30^. It is evident that embryo freezing/thawing is not without significant risk.

We must be aware of a functional link between the interference with epigenetic reprogramming in very early development and adult diseases and its relation with ART techniques. Follow-up studies on children born after ART should be performed throughout their life to monitor their metabolic, cardiovascular, endocrine and weight status.
